# An embodied struggle of visibility in all its invisibility – mothers’ lived experiences of oversupply of breastmilk during breastfeeding in Sweden

**DOI:** 10.1186/s13006-026-00911-z

**Published:** 2026-11-03

**Authors:** Ellinor Mehtola, Marie Golsäter, Lina Palmér

**Affiliations:** 1 Faculty of Caring Science, University of Borås, Work Life and Social Welfare, Borås, Sweden; 2 Child Health Services, Region Jönköping County, Jönköping, Sweden; 3 CHILD--research group, School of Health and Welfare, Jönköping University, Jönköping, Sweden

**Keywords:** Oversupply of breastmilk, Mothers, Breastfeeding care, Abjection, Lifeworld research, Phenomenology

## Abstract

**Background:**

Breastfeeding difficulties can awaken a sense of existential vulnerability. One difficulty is oversupply of breastmilk, which is typically described from a medical perspective, while there is a lack of research on the lived experiences of this and on how it affects mothers existentially. This study’s aim is therefore to describe breastfeeding mothers’ lived experiences of oversupply of breastmilk during breastfeeding.

**Methods:**

A reflective lifeworld research approach was used during the whole research process. Individual lifeworld interviews were conducted with 17 mothers in Sweden with lived experiences of oversupply of breastmilk during breastfeeding.

**Results:**

The results of this study illuminate the meaning of oversupply of breastmilk during breastfeeding as it is experienced by breastfeeding mothers. The essential meaning can be described as *an embodied struggle of visibility in all its invisibility*. This captures the bodily and existential experience of navigating between being seen and unseen, further described in the following constituents: being trapped in a milk-producing body, being powerless in a vulnerable breastfeeding situation, being insufficient as a mother, and struggling in loneliness.

**Conclusions:**

From a caring science perspective, this study contributes important knowledge which can help in developing caring practices. The results highlight that oversupply of breastmilk during breastfeeding is an existential phenomenon, with existential dimensions, that affects a mother’s life in a demanding and challenging way when it comes to being a mother. Here the idea of abjection acts as a barrier, in which the uncontrollability of the breastmilk can create an aversion towards one’s body, one’s breastmilk and breastfeeding. There is a risk that these existential dimensions may remain hidden or unspoken if the mother does not dare talk about it and/or the caregiver does not understand that it needs to be discussed. Lifeworld-led care presents an opportunity in which the lived experience is the focus of the caring and the existential is allowed to come to the fore alongside the medical. The intertwining of these two aspects has the potential to contribute to caring that supports and strengthens health and well-being in an existentially challenging and demanding breastfeeding situation.

## Background

Although pregnant women may wish to breastfeed and can assume it will be easy [1], initiating breastfeeding as a new mother has been described as an unsettled journey [2]. It is marked by an unpreparedness for the situation, and feeling out of control and on one’s own. Breastfeeding is more than just a biological function; it is also an existential issue that creates an embodied relationship between mother and child [3]. If the mother also experiences breastfeeding difficulties this can awaken a sense of existential vulnerability [4] and deep sadness and despair [5] at the fact that the breastfeeding journey didn’t turn out as expected. Oversupply of breastmilk is a breastfeeding difficulty that is typically understood from a medical perspective, and is often underdiagnosed [6]. While symptoms can vary, it is most common for the mother to experience leakage and a strong milk ejection reflex; she can also experience continuous breast pain that can be both sharp and grinding [7]. Problems with latching and pain are a common reason for ceasing breastfeeding [1]. Oversupply of breastmilk is also a risk factor for lactation mastitis [8]. It has been described that mothers experiencing oversupply have reported feelings of frustration and loneliness [9], but without discussing how this affects them existentially. Previous research on oversupply of breastmilk focuses mostly on symptoms [7, 10] and assessment [6, 7, 11] from a more medical perspective. According to Toombs [12], in order to prevent a person from losing their sense of personhood it is necessary to also address their lived experience and not only focus on their medical condition. These insights are based on Merleau-Ponty’s [13] philosophy that not only do humans have a body as an object; we *are* our bodies, and these bodies are lived as a subjective whole in which meaning is created. Rooted in phenomenological philosophy, Todres, Galvin and Dahlberg [14] have developed a lifeworld-led caring science perspective that seeks to understand health, suffering, and well-being through lived experiences, emphasizing openness to the meanings embedded in everyday life. The central base of the lifeworld-led perspective is to develop caring that supports health and wellbeing based on lived experiences of human beings.

From a lifeworld-led caring science perspective, is it therefore important to have knowledge about the lived experience of being a mother experiencing oversupply of breastmilk during breastfeeding, in order to develop caring practice that eases suffering as well as strengthening health and well-being [15]. A caring science approach based on a lifeworld perspective provides a more in-depth understanding of oversupply of breastmilk than medical and diagnostic approaches and methods alone can [16].

To the best of our knowledge, previous research on the phenomenon of oversupply of breastmilk as experienced by breastfeeding mothers is scarce and outdated. Knowledge from the mother’s perspective may lead to an increased understanding from those who provide breastfeeding support, and has the potential to develop caring practice that meets mothers’ existential needs. Therefore, the aim of this study is to describe breastfeeding mothers’ lived experiences of oversupply of breastmilk during breastfeeding.

## Method

### Design

This phenomenological study adopts a reflective lifeworld research (RLR) approach [16]. RLR derives its epistemological basis from phenomenological and hermeneutical philosophy. It entails a phenomenon- and meaning-oriented focus on the lived experience, and is characterised by keeping the phenomenon in focus throughout the entire research process, including data collection and analysis. A phenomenon is ‘something as it is experienced by someone’; in this study, the phenomenon is ‘oversupply of breastmilk during breastfeeding’. To apprehend a phenomenon, a phenomenological attitude is required, that acknowledges the natural attitude, and intentionality, in which attention is directed in order to study the phenomenon in a rigorous manner. To maintain this phenomenological attitude, the methodological principles of RLR emphasise openness, flexibility and bridling the process of understanding as the research unfolds. Openness is achieved through bridling conducted with a reflective attitude and by maintaining flexibility regarding how the phenomenon, here oversupply of breastmilk, manifests within the data. Bridling involves a conscious and ongoing effort to slow down understanding of the phenomenon, critically reflect on pre-understandings as well as understanding of the phenomenon, and remain open to the meanings emerging from participants’ lived experiences in order to describe the phenomenon in a new way. The philosopher Hans-George Gadamer encourages the researcher to see “the otherness” of the phenomenon e.g. what is new and surprising instead of what we already know from our pre-understanding (16). This is continually pursued by asking reflective and critical questions of both the data and oneself – for example, ‘Why do I understand it this way? Could it mean something else?’ [16].

### Participants and Setting

The mothers were invited to participate in the study through advertising in social media, sharing in breastfeeding support groups, and through child health nurses. The inclusion criterion was a lived experience of the phenomenon of oversupply of breastmilk during breastfeeding. The advertisement contained contact information for the first author, and mothers who were interested in participating were asked to communicate this via email. After initial contact, all interested mothers received a response email with further information about the study. Those who continued to express interest were subsequently sent a formal information letter. In total, 23 mothers maintained their interest beyond this stage, and 17 of them agreed to participate in interviews. In accordance with phenomenological methodology and RLR [16], recruitment aimed to obtain rich and nuanced descriptions of the phenomenon in terms of lived experience. Data collection was concluded when the material reflected a broad and varied range of meanings, providing sufficient depth to illuminate the essential structure of the phenomenon.To ensure variation in how the phenomenon was experienced, strategic sampling was applied based on the mother´s age, number of children, country of origin, and the time elapsed since the experience, see Table 1. Recruitment concluded when the material was judged to have reached meaning saturation. Even after this point, additional mothers continued to express interest, resulting in a total of 74 mothers who made contact. The included mothers’ ages varied between 28 and 49 years, number of children between one and four, and time since the experience between having the situation right now and 22 years ago, the majority of the mothers having children younger than five years of age. The interviews were conducted in Sweden between November 2023 and March 2024.

**Table 1:** Demographic characteristics of participants

Age	Mothers´(*n* = 17)
20–29	1
30–39	13
40–49	3
**Number of Children**	
1	8
2	6
3–4	3
**Country of birth**	
Sweden	15
Outside Sweden	2
**Time elapsed since first time breastfeeding**	
0–1 year	8
2–5 year	6
6–22 year	3

### Data collection

In order to describe the lived experience of the phenomenon of oversupply of breastmilk during breastfeeding as experienced by mothers, lifeworld interviews were conducted in accordance with RLR [16]. Lifeworld interviews are phenomenon- and meaning-oriented, focusing on the lived experience of a specific phenomenon. The methodological principles of RLR – openness, pliability and bridling – were practised throughout the interviews. In this way, the phenomenon was explored with an open and reflective attitude towards it. Each interview started with an open request to talk about the phenomenon: ‘Can you please talk about your experiences of oversupply of breastmilk?’ This allowed for openness and pliability towards the phenomenon. To obtain an in-depth description of the phenomenon, participants were encouraged to express their experiences in an open and reflective manner, facilitating bridling of the phenomenon. The phenomenon was further explored during the interview through follow-up questions. Examples include the following: ‘Can you tell me a bit more?’ ‘How did that make you feel?’ ‘What did this mean for you?’. The 17 interviews were conducted by the first author. Participants could choose between face-to-face, videoconference, or telephone interviews. However, no participant selected the face-to-face option. Fourteen mothers chose to participate via videoconference, and three opted for a telephone interview. The interviews lasted between 30 and 82 min, with an average length of 48 min. The individual interviews were recorded only on audio file and transcribed verbatim.

### Data analysis

The analysis followed the RLR principles, in accordance with Dahlberg, Dahlberg and Nyström [16]. The process was characterised by movement between the whole (the interviews) and its parts (meaning units), and then towards a new whole (the essence). Throughout these movements, the aim was to remain as open and reflective as possible, adopting an approach marked by closeness to the text and a flexible and open attitude to its meanings. This was undertaken in order to prevent researchers’ preconceptions from prevailing and thereby determining in advance what the participants meant. First, all authors read the transcribed interviews separately and repeatedly to obtain an understanding of them as a whole. Subsequently, an analysis of the parts commenced, seeking meanings related to the phenomenon of oversupply of breastmilk during breastfeeding. These meanings formed patterns consisting of clusters, in which related meanings were linked. The creation of clusters was an ongoing process of reflecting process among all authors, exploring different possibilities in search of the essential structure of meanings in relation to the phenomenon. All authors were consequently involved in all parts of the analysis. The essential meaning of the phenomenon – its essence – constitutes the most abstract level, describing how the meanings across all identified clusters relate to one another; i.e., the invariant meaning of the phenomenon. The essential meaning consists of constituents, variations in meanings on a more contextual level. The constituents of meanings further illuminate the essential meaning, and these constituents are exemplified using quotes from the interviews [16].

### Ethical consideration

This study was conducted according to the research principles described in the Declaration of Helsinki [17] and follows Swedish research ethics guidelines (SFS 2003:460) [18]. Ethical approval was sought and received from the Swedish Ethical Review Authority (Dnr: 2023-02849-01). Both written and oral information on the study’s aim was given to the participants, and it was emphasised that they had the right to cease participation at any time, even during the ongoing process of analysing. To ensure participants’ integrity and compliance with ethical guidelines [18], the interviews were pseudonymised by the first author who conducted the transcription. Only pseudonymised transcripts were shared within the research team. Identifying information is stored separately, in coded form, and secured according to established ethical and data-protection standards.

## Results

The essential meaning of the phenomenon of oversupply of breastmilk during breastfeeding can be described as *an embodied struggle of visibility in all its invisibility*. This captures the bodily and existential experience of navigating between being seen and unseen, profoundly influencing a mother’s sense of being in the world. Being visible in all its invisibility signifies that motherhood is fundamentally challenged by the significant bodily presence of breastmilk. This challenge emerges as breastmilk creates a barrier – both physical and existential – between the mother, the child, and the world.

The embodied struggle of being seen and unseen entails a paradoxical presence – being physically and visually present while remaining invisible in one’s existential needs and emotions. This struggle underlines the complexities of breastfeeding and the journey into motherhood. The embodiment manifests itself as a deeply anchored connection to the world, the self, and others, in which the breastmilk profoundly shapes one’s presence and relationships in a demanding and complex way. The bodily reality of breastmilk influences both the mother’s existence and her relationship with her child, deeply impacting their interconnected experiences.

Attempts to conceal the uncontrollability of the body render the mother invisible to others, while her entire existence silently demands to be seen as an existentially vulnerable mother in need of being cared for. The embodied struggle of being visible in all its invisibility signifies a loss of agency as the body and breastmilk take over, exposing the mother in a way she does not want. This visibility blurs the boundaries of embodiment, as the oversupply of breastmilk threatens the mother’s sense of integrity. The visibility of the breastmilk invites trespasses on her boundaries by others, leading to a vulnerability that challenges her existence. Being invisible, on the other hand, diminishes the mother’s presence, as her experiences are dismissed by others and she may even devalue herself. The phenomenon of oversupply of breastmilk during breastfeeding thus reveals a profound tension: a continuous negotiation of presence, being and boundaries in the embodiment of motherhood.

The essential meaning *an embodied struggle of visibility in all its invisibility* is further described in the following constituents: being trapped in a milk-producing body, being powerless in a vulnerable breastfeeding situation, being insufficient as a mother, and struggling in loneliness.

### Being trapped in a milk-producing body

Oversupply of breastmilk during breastfeeding means being trapped in a milk-producing body without the possibility to escape. One’s bodily boundaries are transgressed by uncontrollable factors, such as excessive leakage of breastmilk; i.e., one’s bodily expressions are visible to everyone in one’s surroundings. The body becomes alienated, with pain, leakage and feelings of ‘unfreshness’ taking over. Due to the leakage, feelings of discomfort arise. *’It’s like living in an ocean of breastmilk’*. Everything is soaked with milk. Feeling disgust for one’s own body emphasises being trapped in a milk-producing body:

I feel a little gross about this, because I think breastmilk smells gross// and even though I’ve showered, milk is still flowing, so it’s a bit difficult to stay clean. (Olivia, mother of one, breastfeeding currently)

Being trapped in a milk-producing body means living with shame over one’s visible bodily expressions. This means that the body becomes one’s enemy, which one does everything to hide from the staring eyes of others. This is done either by avoiding breastfeeding in public or making sure in every situation to have supplies to handle the leakage of one’s milk-producing body, in order to feel secure. These actions are done in secret. If the oversupply of breastmilk shows in public it places the mother in the spotlight around strangers, making her highly vulnerable:

You’re somewhere among people and feel like the milk ejection is starting – damn, I’ll have to go and change soon // then you have to simply excuse yourself and go away to change, and I’ve brought identical clothes with me completely unnecessarily // so that there wouldn’t be anything obvious about me that made it not perfect. (Edith, mother of two, breastfeeding in the past and currently)

Being trapped in a milk-producing body also limits one’s possibilities to live a life outside the home. In this situation the home becomes an ambiguous place, simultaneously a safe place and a prison. The home as a safe place is where there are ways to manage the oversupply of breastmilk in the breastfeeding situation without being exposed by the gaze of others; meanwhile, the home as a prison means that a mother’s milk-producing body forces her to stay at home, leaving her being locked in and trapped:

I needed to have my nursing station at home with extra nursing pads, extra everything // it became so very tied to the stations I had at home // I didn’t feel particularly locked in because of the baby, but I felt locked in because of my oversupply. (Thea, mother of two, breastfeeding in the past and currently)

Being trapped in a milk-producing body without the possibility to escape also means living with despair in a hopeless breastfeeding situation, which forces one to negotiate between enduring and giving up. Negotiation is necessary on the journey of becoming a mother on one’s own terms. In becoming the breastfeeding mother one desires to be, it is necessary to escape from one’s own emotions and mentally endure, despite one’s whole body protesting and wanting to leave the situation.

### Being powerless in a vulnerable breastfeeding situation

Oversupply of breastmilk during breastfeeding means being powerless in a vulnerable breastfeeding situation. This entails a loss of agency whereby the oversupply of breastmilk controls every aspect of one’s existence, resulting in fatigue and resignation in an uncontrollable life situation:

…very broken on all levels. Cognitively there was nothing left, physically there was nothing left, emotionally it’s completely drained // it was survival mode on all levels. (Charlotte, mother of one, breastfeeding in the past)

Being powerless in a vulnerable breastfeeding situation illuminates a decreased confidence, in which feeling insecure as a mother doubting one’s own ability to breastfeed dominates one’s life situation. This creates insecurity and feelings of guilt, which are reinforced when those around one don’t understand or see the difficulty with oversupply of breastmilk:

This guilt, you feel like this is something I should’ve handled // because I noticed myself that it wasn’t good and that the baby wasn’t feeling really well// while no one else thought it was a problem // it becomes a strange conflict within oneself // how can it be like this – if there’s nothing wrong then how can it be this hard? (Fiona, mother of three, breastfeeding in the past)

In such a powerless situation, becoming a mother is hard to manage. The motherhood one imagined, with breastfeeding being a significant aspect, now loses its direction, in terms of a lost way into motherhood without a purpose. This includes difficulties navigating between one’s own image of being a mother who can mother her child with comfort and joy on the one hand, and simply being reduced to a milk machine on the other.

### Being insufficient as a mother

Oversupply of breastmilk during breastfeeding also means being insufficient as a mother, in which the relationship with one’s child is hindered due the bodily discomfort. This discomfort manifests itself as a hindrance when it gets in the way of acts of tenderness and comforting the child that do not involve eating. Feeling that one’s own body is causing one’s child pain, discomfort and harm is overwhelming. This creates feelings of guilt, and causes mothers to blame themselves for their child having a hard time when breastfeeding:

My son was about six days old when he choked and his whole body went limp // it was very, very hard // when he choked and went completely limp, I thought he was going to die // as a mother, you think you should do the best for your child // I still feel guilty that I could hurt my child or that he might not find breastfeeding as enjoyable. (Tina, mother of two, breastfeeding in the past and currently)

Being insufficient as a mother means having a disbelief in one’s own ability in the breastfeeding situation; one thinks one should be able to handle the situation, but in this instance motherhood becomes difficult:

You don’t solve the problem; the problem is that the baby’s hungry and I try to feed him, but milk spurts all over and I can’t make it work, the baby can’t eat what he should and he chokes, coughs and cries // he doesn’t get satisfied and then you feel inadequate. (Susie, mother of one, breastfeeding currently)

Being insufficient as a mother also involves a fear of how it will affect one’s child in the future: whether the difficulties caused by the oversupply will persist as they get older. This fear creates anxiety that the motherly care of breastfeeding one’s child will create lifelong suffering for them, with lasting effects such as persisting stomach pain, disturbed bacterial flora and eating difficulties:

It’s hard to know how serious it is, if it’s a big problem or if it’s a small one // is it something that will have effects on her development // will she have stomach pains for the rest of her life. (Tess, mother of one, breastfeeding currently)

Being insufficient as a mother also means feeling anger and frustration towards both one’s child and the situation. The anger towards one’s child manifests itself as blaming them for not eating properly, which itself creates feelings of guilt. This makes one doubt one’s own ability as a mother:

…so I could only feel but why can’t you just eat like a normal ordinary child, the feeling’s a bit like this and it feels completely macabre to think like this about your six-week-old baby, but please // there’s plenty of food, can’t you just eat// it’s a terrible thought to put that responsibility on the baby, but I did. (Elsa, mother of three, breastfeeding currently and in the past)

Being insufficient as a mother also means feeling sadness about the fact that the breastfeeding did not turn out as desired; this creates a longing to succeed at it. This longing to achieve the desired breastfeeding and become the mother one has imagined creates a resistance to giving up despite the obstacles that the oversupply creates.

### Struggling in loneliness

Oversupply of breastmilk during breastfeeding also means struggling in loneliness, being left alone with one’s own worries and imagining being the only mother in the world experiencing this. Believing that one is alone with these difficulties causes one to keep silent about one’s experiences, while longing to share one’s breastfeeding story. This longing entails a desire to meet a genuinely curious other who is there and listens without prejudice. Without someone listening to one’s breastfeeding story, one’s already difficult everyday life is almost unbearable and the struggling in loneliness deepens. The belief that one is alone entails feelings of being abnormal and odd:

It felt lonely because they said it would go away after a certain amount of time and it took more than twice that, so I felt like I didn’t dare tell them much because then everyone would say ‘but stop breastfeeding then’; I thought he deserved my trying, that I fought // Oh but I didn’t want to stop breastfeeding. I just wanted to find a way so I could breastfeed so it worked for both of us so he could experience it and then I found it, but it cost me a lot (crying). (Nina, mother of two, breastfeeding in the past)

Struggling in loneliness means diminishing and hiding one’s own feelings, often in consideration of those around one. This includes being met with misunderstanding from others, which leads one to withdraw from social interactions. There is a conflict between one’s own experiences and the perceptions of others. Oversupply is described by others as a first-world problem; this description does not fit the mothers’ experience, however, and makes them furious at being met with misunderstanding, causing them to feel despair over their existence:

It may sound like a luxury problem // it wasn’t luxurious; we weren’t glamourous. (Sarah, mother of one, breastfeeding currently)

Struggling in loneliness also means being forced to search for information and solutions by oneself, when healthcare providers offer no evidence-based information. Furthermore, it means being ignored when seeking help; this involves being misunderstood, trivialised and questioned. This ignoring among healthcare professionals appears when they focus on the child’s growth chart, without being open to the mother’s breastfeeding story:

It can’t just be how the points are on the chart, that is, the results; it has to be about the journey to the chart and being seen on that journey. I think it was really hard to be heard // he seemed chubby and healthy, whatever the cost is for the mother. (Elsa, mother of three, breastfeeding currently and in the past)

Struggling in loneliness also involves being deeply saddened at being alone in an unsustainable existence, with one’s whole self, crying out to be made visible inside. This creates a longing to be made visible as a struggling mother, and to have it confirmed that one’s motherhood is good enough.

## Discussion

The aim of this study is to describe breastfeeding mothers’ lived experiences of oversupply of breastmilk during breastfeeding. The essential meaning shows that the phenomenon of oversupply of breastmilk during breastfeeding is experienced as an embodied struggle of visibility in all its invisibility. This can be seen as a struggle to make the visible (such as being trapped in an uncontrolled body, with bodily leakage and strong milk ejection) invisible, and the invisible (loneliness, struggle, vulnerability and fear of hurting one’s child) visible. This captures the bodily and existential experience of being in an exposed breastfeeding situation, navigating between being seen and unseen, that deeply influences a mother’s sense of being in the world as a mother. Taken together, in a caring situation the presence of oversupply of breastmilk seem to acts as a barrier and threatens to hide mothers’ existential needs; instead, the focus seems to be on the child`s needs, and the child`s weight gain. This has also been shown in research by Hamnøy, Kjelsvik, Baerung and Dahl [19], who found that breastfeeding mothers want to been seen as a whole, and for their breastfeeding difficulties to be taken seriously. It is thus important to highlight this situation in terms of developing caring practices that take into account both medical and existential perspectives, with attention to the lived experiences of mothers. In this respect, mothers experiencing an oversupply of breastmilk do not differ from other mothers with breastfeeding difficulties [4, 5, 20, 21], as the lived experience of navigating strain, imbalance, and embodied disruption tends to unfold in similar ways across these situations and remains out of step with the imagined horizon of what it means, in their lifeworld, to be the breastfeeding mother they long to be.

Julia Kristeva’s [22, 23] theory of abjection helps us understand that the visibility of breastmilk disturbs identity and disrupts order in a society in which women’s bodies should be invisible and behave in an objective and clear manner. This theory describes phenomena that evoke discomfort, disgust or unease because they transgress boundaries between self and other, body and culture, subject and object. The abject is something that is both part of the self and yet not the self, creating a sense of ambiguity and difficulty in locating it within one’s identity. In relation to women’s bodies, abjection often concerns biological processes that are fundamentally human but culturally regulated – such as menstruation blood, secretions from the vagina and, in relation to this study, breastmilk. For women with oversupply of breastmilk, this relates to the experience of breastmilk as excessive and uncontrollable: something that leaks, drips or sprays, something they cannot fully regulate, control or keep ‘within boundaries’. Breastfeeding that might be framed as natural, beautiful and maternal is shown in the present study to instead be experienced as disturbing, shameful or alienating, making the body feel unfamiliar or out of control. In this way, the oversupply of breastmilk represents a form of the abject: bodily fluid from a woman that, when present in excess, generates worry, discomfort or even disgust – both in the woman herself and particularly in those around her. This is one way to explain why the presence of breastmilk is threatening to a mother’s identity and is therefore rejected by others, such as society and in worst case also by healthcare professionals.

This study also highlights existential loneliness and struggle due to abjection. One concrete example of this is mothers’ experiences of not being met with respect and not being taken seriously, their voices instead being silenced. This makes the situation an invisible difficulty. Fears of harming one’s own child due to the cascade of breastmilk are evident in everyday life as a mother, which is stressful and existentially draining. This study’s results show that the mothers’ feelings towards themselves and the breastmilk leakage are those of shame, disgust and unfreshness.

According to Kristeva, abjection leads to alienation and to becoming a foreigner in one’s own body [22, 23], feeling like an outsider and being excluded or ignored by others. The mothers in this study struggle to hide the visibility of their oversupply not only in public but also in the company of family and friends. Breastfeeding in public is itself associated with stigma [24], even though mothers wish it were seen as the norm and were thereby accepted to do openly in public [25]. In art and pictures [26] as well as in research [27], breastfeeding is often viewed as a harmonic albeit isolated dyad between mother and child [26], and when the experience of breastfeeding does not correspond with this image it creates a sense of shame and failure as a mother. Bottle-feeding mothers [28] and mothers with primary low milk supply [29] experience a similar kind of shame and failure when breastfeeding doesn’t match their imagined harmonic picture. However, experiencing oversupply of breastmilk adds another layer to this, and the fear of causing a scene hinders the mothers from taking part in society even more.

As a result of the idea of abjection, with the mother hiding the oversupply of breastmilk, she becomes invisible in her struggle to overcome the difficulties she is experiencing with breastfeeding. Paradoxically, this study shows that this is true even though the mother wishes to be seen and confirmed in her existentially exposed situation. The duality of those around the mothers cheering and encouraging them to be grateful on the one hand and the experience of pain, discomfort and fatigue on the other creates an internal conflict, with feelings of both gratitude and shame. Other studies highlight the positive side of excessive breastmilk supply in terms of donation, with mothers feeling gratitude at being able to give their extra milk to those in need [30, 31].

Previous studies emphasise that breastfeeding mothers want to have updated advice and personalised care [19, 32]. Being listened to and seen as a struggling woman, and being met in one’s existential needs, is crucial to creating breastfeeding care that is caring [33]. This study’s results, and the discussion related to Julia Kristeva’s theory of abjection [22, 23], deepen the understanding of why experiencing oversupply of breastmilk leads to feelings of loneliness, frustration and social isolation that has previously been reported by Wilson-Clay [9].

Merleau-Ponty’s [13] philosophy helps us see a person as being-in-the-world as a lived body, with experience and body profoundly intertwined. With this understanding it becomes clear that the bodily physical, emotional and existential challenges brought about by breastfeeding difficulties such as oversupply of breastmilk cannot be separated but rather need to be addressed as a whole; i.e., the mother being met as a whole person. This can be achieved if caregivers have an understanding of and openness to the mother’s ‘insiderness’ due to her lived experiences, being open to what the experiences are like for her [34].

In order to develop caring practice that enables health and well-being, the mother’s lived experiences need to be highlighted in the meeting with her, and the bodily and existential issues balanced [12, 35]. The caregiver must also in every encounter dare to see the otherness [36] in terms of what is new and significant for the individual breastfeeding mother; otherwise the preunderstanding, the things we already know, risk obscuring the view. From a caring perspective, it is important to have a reflective and open attitude when caring for mothers with oversupply of breastmilk, in order to not reinforce the abjection. Instead, when caring for mothers who are breastfeeding, caregivers must be able to visualise and talk about how abjection might affect the mother’s experience, thereby giving them the acknowledgement they need.

### Methodological reflections

To ensure objectivity, transferability and validity, the study followed the RLR approach [37]. A phenomenological analysis was conducted according to RLR, which was considered a suitable approach for describing lived experience, and also contributed to the study’s validity [16, 37]. By maintaining an open, bridling and reflective attitude, highlighting our preunderstanding and actual understanding of the phenomenon, objectivity was achieved [16]. During data collection this was accomplished by asking reflective questions in order to slow down the understanding process. The aim of describing the essential meaning of the phenomenon involves obtaining knowledge that goes beyond individual descriptions and providing an essential meaning structure instead of dividing meaning into separate categories. In this lies transferability, which is a core strength of phenomenological studies [16]. It is also through the essential meaning that validity is achieved, as the aim is to try to understand what the meaning of a certain phenomenon is, rather than merely referring to what people say or do [37].

The participants were free to choose how the interviews were conducted, face-to-face, video conference or telephone, and this adaptation to their wishes contributed to their feeling safe and more confident in the environment where the interviews were held [16]. Previous research [38] has shown that distant interviews, such as via video conference or telephone, can enhance participants’ sense of security and anonymity, thereby increasing their willingness to share their lived experiences.

In line with RLR, variation among participants was sought to capture different expressions of the phenomenon and to illuminate its meaning from multiple experiential perspectives [16]. Such variation contributes to a richer understanding of the phenomenon, supporting the exploration of both its essential meanings and the ways in which these meanings may be manifested across different life situations and timepoints of experience. In the present study, there was variation in the temporal distance to the experience, ranging from women currently experiencing oversupply to those reflecting on experiences that had occurred up to 22 years earlier.

Reflections during the interviews showed that the embodied memory of the phenomenon remained vivid; participants could, for instance, begin to cry despite having experienced it more than two decades earlier. Merleau-Ponty [13] describes this as the body remembering through its embodied intentionality. Events that are central to one’s life remain central regardless of the time that has passed [39].The chosen RLR approach [16] is well suited for phenomenological studies, in which the focus is on the phenomenon and not the individuals; all of the interviews included rich and varied content that described the phenomenon. This study contributes to a better understanding of having oversupply of breastmilk while breastfeeding, as described by the mothers themselves.

### Limitations

The findings are grounded in the lived experiences of a specific group of mothers in Sweden and should therefore be understood within their particular cultural and experiential context. Given the small sample, the single cultural setting, and the variation in participants’ temporal distance to the experience of oversupply, the findings cannot be generalized to all mothers with an oversupply of breastmilk. Nevertheless, the identified essence strengthens the validity of the findings by illuminating how the meaning of the phenomenon may be transferable to other than participating mothers who experience oversupply of breastmilk during breastfeeding. Although variation in participants’ experiences was sought and is considered a strength in phenomenological research, there was substantial variation in the temporal distance to the phenomenon, ranging from women currently experiencing oversupply to women reflecting on experiences that had occurred up to 22 years earlier. While such variation may enrich the understanding of the phenomenon through different experiential perspectives, it may also have influenced participants’ recollections and descriptions of their experiences.

Limitations can also be related to the data collection procedure. Some interviews were conducted by telephone or video conference, which entailed a risk that non-verbal expressions could be missed during the interview. At the same time, these interview formats enabled the inclusion of participants from a wider geographical area and thereby contributed to greater variation in the material. To minimise the risk of losing important non-verbal information, such as crying, sighing, or hesitation, the same author who conducted the interviews also performed the transcriptions.

Additional limitations may be related to the inclusion criteria and shared characteristics of the participants. All participants were committed to continuing breastfeeding despite their experiences of oversupply. Consequently, some variations of the phenomenon may not have been captured, particularly the experiences of mothers who discontinued breastfeeding early because of severe difficulties related to an oversupply of breastmilk. This may have limited the range of meanings illuminated in the present study.

## Conclusion

The main findings and the essential meaning of the phenomenon of oversupply of breastmilk during breastfeeding can be described as *an embodied struggle of visibility in all its invisibility*. From a caring science perspective, this study contributes important knowledge that can help breastfeeding care come to enable health and well-being for each individual mother. The results highlight existential dimensions of the phenomenon of oversupply of breastmilk: being trapped, lonely, powerless and insufficient as a mother. But these dimensions may remain hidden or unspoken if the mother does not dare talk about it and/or the caregiver does not understand that it needs to be discussed. Here the idea of abjection acts as a barrier, in which the uncontrollability of the breastmilk can create an aversion towards one’s body, one’s breastmilk and breastfeeding. The lifeworld-led care presents an opportunity in which the lived experience is the focus of the caring and the existential is allowed to come to the fore alongside the bodily/medical. The intertwining of these two aspects has the potential to contribute to caring that supports and strengthens health and well-being in a challenging breastfeeding situation.

The results imply that there are deficiencies in the caring practices for mothers with oversupply of breastmilk. This needs to be further explored in order to develop care that is caring and meets the existential needs of mothers with oversupply of breastmilk.

## Data Availability

The data generated and analysed during the current study are not publicly available due to privacy and ethical restrictions, but are available from the corresponding author on reasonable request.
